# Self-Reported Medication Use among Pregnant and Breastfeeding Women during the COVID-19 Pandemic: A Cross-Sectional Study in Five European Countries

**DOI:** 10.3390/ijerph19031389

**Published:** 2022-01-26

**Authors:** Michael Ceulemans, Veerle Foulon, Alice Panchaud, Ursula Winterfeld, Léo Pomar, Valentine Lambelet, Brian Cleary, Fergal O’Shaughnessy, Anneke Passier, Jonathan Luke Richardson, Hedvig Nordeng

**Affiliations:** 1 Clinical Pharmacology and Pharmacotherapy, Department of Pharmaceutical and Pharmacological Sciences, KU Leuven, 3000 Leuven, Belgium; veerle.foulon@kuleuven.be; 2 Teratology Information Service, Pharmacovigilance Centre Lareb, 5237 MH Hertogenbosch, The Netherlands; a.passier@lareb.nl; 3 L-C&Y, KU Leuven Child & Youth Institute, 3000 Leuven, Belgium; 4 Service of Pharmacy, Lausanne University Hospital and University of Lausanne, 1011 Lausanne, Switzerland; alice.panchaud@chuv.ch; 5 Institute of Primary Health Care (BIHAM), University of Bern, 3012 Bern, Switzerland; 6 Swiss Teratogen Information Service, Clinical Pharmacology Service, Lausanne University Hospital, 1011 Lausanne, Switzerland; ursula.winterfeld@chuv.ch; 7 Materno-Fetal and Obstetrics Research Unit, Lausanne University Hospital, 1011 Lausanne, Switzerland; leo.pomar@chuv.ch (L.P.); valentine.lambelet@gmail.com (V.L.); 8 School of Health Sciences (HESAV), University of Applied Sciences and Arts Western Switzerland, 1011 Lausanne, Switzerland; 9 Rotunda Hospital, D01 P5W9 Dublin, Ireland; bcleary@rotunda.ie (B.C.); foshaughnessy@rotunda.ie (F.O.); 10 School of Pharmacy and Biomolecular Sciences, Royal College of Surgeons in Ireland, University of Medicine and Health Sciences, D02 VN15 Dublin, Ireland; 11 UK Teratology Information Service, Newcastle upon Tyne Hospitals NHS Foundation Trust, Newcastle upon Tyne NE2 4AB, UK; jonathan.richardson3@nhs.net; 12 Pharmacoepidemiology and Drug Safety Research Group, Department of Pharmacy, Faculty of Mathematics and Natural Sciences, University of Oslo, 0316 Oslo, Norway; h.m.e.nordeng@farmasi.uio.no; 13 Department of Child Health and Development, Norwegian Institute of Public Health, 0213 Oslo, Norway

**Keywords:** pregnancy, lactation, breastfeeding, medication use, drug utilization, pharmacoepidemiology, COVID-19, SARS-CoV-2, pandemic, Europe

## Abstract

Insight into the epidemiology of perinatal medication use during the COVID-19 pandemic is scarce. Therefore, a cross-sectional study using an anonymous web survey was performed in Ireland, Norway, Switzerland, The Netherlands, and United Kingdom (UK) to investigate the prevalence and type of medications used by pregnant and breast-feeding women during the first pandemic wave. Factors associated with medication use were estimated by logistic regression. In total, 8378 women participated (i.e., 3666 pregnant and 4712 breastfeeding women). Most responses were collected in Norway (34%) and The Netherlands (28%), followed by Switzerland (19%), Ireland (17%) and UK (2%). Participants were more often professionally active and more often had a higher educational level compared to the general birthing population in each country. Overall, approximately 60% of women reported having used at least 1 medication in the preceding 3 months. Daily and occasional use was reported by 34% and 42% of pregnant and 29% and 44% of breastfeeding women. The most prevalent ATC (Anatomical Therapeutic Chemical) categories were the nervous system, the respiratory system, the alimentary tract/metabolism, and the musculo-skeletal system. Paracetamol, ibuprofen, antacids, and cetirizine were the most frequently used medications. The rate of antibacterial use was lower than previously reported. Having a chronic illness, country, maternal age, SARS-CoV-2 testing, professional status and time since delivery were associated with medication use. In conclusion, perinatal medication use was highly prevalent during the first pandemic wave, underlining the importance of maintaining counseling efforts on medication use, even in times of disrupted healthcare services and/or limited resources.

## 1. Introduction

The 2019 novel coronavirus pandemic and its related disease (COVID-19) has obviously had a substantial impact on peoples’ professional and recreational lives [1,2]. At the same time, the containment measures imposed by governments to reduce virus transmission have disrupted patients’ access to healthcare services and medical follow-up, including among pregnant and breastfeeding women [3,4,5]. Disrupted access to healthcare services, along with the potential adverse effects of COVID-19 in pregnancy, may have impacted the physical wellbeing of pregnant and breastfeeding women [6]. Besides the increased risk of physical sequelae, studies have shown the potential adverse impact of the pandemic on women’s emotional wellbeing with increased prevalences of perinatal depression and anxiety [7,8,9].

The potential COVID-19 related adverse effects on physical and emotional wellbeing might have influenced medication use within the general public, including among pregnant and breastfeeding women. Perinatal drug utilization studies performed before the pandemic have shown that medication use in pregnancy is very common, with up to 80% of women using at least 1 prescription medication during their pregnancy [10,11,12,13]. Medication exposure also frequently occurs during breastfeeding and in the postpartum period with at least 50% of women using a medication [14,15,16]. Although numerous perinatal drug utilization studies were undertaken prior to the COVID-19 era, no data are available from studies performed during the pandemic. Nevertheless, several papers have raised awareness for or provided evidence on the potential shifts in drug utilization patterns during the pandemic. Examples of this include the publication of premature assumptions on the beneficial or detrimental effects of medications on COVID-19 outcomes (e.g., hydroxychloroquine, non-steroidal anti-inflammatory drugs) [17,18]. Other publications have shown that some medications have been used or dispensed to a lesser extent within the general public (i.e., antibiotics, insulin) [19,20,21], whereas other medications have been prescribed or dispensed more often (i.e., psychotropics and antidepressants) [22,23,24]. However, the extent to which this phenomenon is applicable to pregnant and breastfeeding women remains unclear.

Overall, drug utilization studies have the potential to provide crucial insight for healthcare professionals (HCPs) and policymakers into the current challenges and opportunities related to medication use. Such insights may be even more valuable during societal and health crises such as the COVID-19 pandemic. In response to this need for knowledge, we set up a multinational COVID-19 study to investigate the prevalence and type of medications used by pregnant and breastfeeding women living in several European countries during the first wave of the pandemic.

## 2. Methods

### 2.1. Study Design and Population

This cross-sectional drug utilization study is part of a multinational project aimed at characterizing pregnant and breastfeeding women’s medication use, COVID-19 vaccine willingness, mental health status, perinatal experiences and practices, and access to health services during the first wave of the COVID-19 pandemic [4,25]. The study was performed in Ireland (IE), Norway (NO), Switzerland (CH), The Netherlands (NL), and the United Kingdom (UK) between 16 June and 14 July 2020. Pregnant women and women who breastfed in the 3 months preceding the survey and who were older than 18 years of age were eligible to participate. Ethical approval was not required in Norway, Switzerland, The Netherlands and the UK as the survey was anonymous and the responses could not be traced back to any of the respondents. In Ireland, the Rotunda Hospital Research Ethics Committee reviewed and approved the survey (REC-2020-017). All of the participants provided online informed consent prior to survey initiation. The results are reported according to the STROBE guidelines [26].

### 2.2. Data Collection

Data were collected through an online survey. The survey was promoted on websites, social media accounts and forums commonly visited by pregnant women and/or new mothers. Information on recruitment tools and internet penetration rates has been summarized elsewhere [25]. The survey was available in English, Norwegian, Dutch, French, German, and Italian (as the latter three are official languages in Switzerland); the English version of the survey relevant to this manuscript is included in the Supplementary Materials (see Figure S1). Detailed information on pilot-testing and survey translation has been reported earlier [25].

### 2.3. Medication Use

Women reported the use of all ‘medicinal products’ in the three months prior to the study. Women were asked to name each product and to indicate whether the use was daily or occasional. To capture attitudes towards medication use, women were asked if they had used a medication during pregnancy or breastfeeding on their own initiative (i.e., without advice of an HCP) and if they were more likely to take medications during pregnancy or breastfeeding due to the coronavirus pandemic. The latter question was rated on a 4-point Likert scale ranging from strongly agree to strongly disagree.

Categorization and coding of all of the recorded medicinal products was performed by national study coordinators using uniform coding instructions. All of the medicinal products were classified into the following six categories: ‘medications’, ‘folic acid’, ‘multivitamins’, ‘iron-containing preparations’ (irrespective of the amount of iron or requirement of a prescription), ‘omega-3 fatty acids’, and ‘other products’. The group ‘other products’ consisted of all types of health products that could not be classified elsewhere, including but not limited to pre- and probiotics, herbal remedies and homeopathic products. After initial categorization, medications were further classified according to the WHO Anatomical Therapeutic Chemical (ATC) classification system [27]. If no route of administration was reported for a medication available in different administration forms, its route was coded as the generally most frequently used route during pregnancy or breastfeeding. Products consisting of more than one active ingredient were categorized as different single medications unless a specific ATC code was available.

### 2.4. Sociodemographic and Health and Reproductive Characteristics

Information on sociodemographic characteristics included country, maternal age, relationship status, professional status, highest education level and smoking in pregnancy/breastfeeding. Information on health and reproductive characteristics included chronic illnesses (i.e., an illness already existing prior to pregnancy), parity, planned pregnancy, gestational trimester at study enrollment, current breastfeeding duration and previous breastfeeding experience. Women were also asked whether they had been tested for SARS-CoV-2 and about the test result. The Edinburgh Depression Scale (EDS) was used to assess the level of depressive symptoms, with a total score of 13 as cut-off for major depressive symptoms [28,29]. Education level was categorized into low, medium, and high level according to national definitions. All of the women who participated in the breastfeeding survey, including women who recently stopped breastfeeding, were considered as ‘breastfeeding women’.

### 2.5. Data Analysis

Descriptive statistics were used to analyze any, daily and occasional use of medications, multivitamins, folic acid, iron, omega-3 fatty acids and other products, and were presented as absolute numbers and percentages. Any medication use during pregnancy and breastfeeding was presented according to women’s sociodemographic, health and reproductive characteristics. The total and average number of medications used daily, occasionally, and overall were calculated. The percentage of women using a medication daily, occasionally, and overall was calculated on both ATC level 1 and 2. Results on the question whether women were more likely to take medications due to the coronavirus were dichotomized into (strongly) agree and (strongly) disagree. Factors associated to any medication use during pregnancy and breastfeeding were estimated by univariable and multivariable logistic regression and presented as crude (cOR) and adjusted odds ratios (aOR) and 95% confidence intervals (CI). All of the available sociodemographic, health and reproductive characteristics were entered as categorical variables in the models. Only significant covariates were retained in the final adjusted models (*p* < 0.05). Multicollinearity of covariates included in the adjusted models was checked. All survey responses were included in the analysis. Statistical analyses were performed with SPSS Statistics version 27 (IBM Corp, Armonk, NY, USA).

## 3. Results

### 3.1. Characteristics of the Participants

In total, 8378 women participated in the survey (i.e., 3666 pregnant and 4712 breastfeeding women), of which 87% completed the survey (i.e., 3183 pregnant and 4077 breastfeeding women). Most responses were collected in Norway (34%) and The Netherlands (28%), followed by Switzerland (19%), Ireland (17%) and the UK (2%). About 90% of the women were professionally active, of which 1/3 in healthcare. A chronic illness was reported by 19% and 16% of pregnant and breastfeeding women. Allergy (5%), asthma (4%) and hypothyroidism (2%) were the most commonly reported chronic illnesses. Depression was reported by 1% of the women, while 15% showed having major depressive symptoms (EDS ≥ 13). Details of participants’ mental health status have been previously reported [25]. Most women were in the third gestational trimester (67%) and ongoing pregnancies were unplanned in 20% of the cases. Overall, 92% of the respondents of the breastfeeding survey were still breastfeeding at the time of study completion. Half of the breastfeeding respondents were between 6 weeks and 6 months postpartum (see Table 1). A comparison with national birthing statistics has been published elsewhere [25], highlighting that participants were more often first-time mothers, higher educated, professionally active, non-smokers, and more likely to report having a partner.

### 3.2. Prevalence of the Use of Medications and Other Health Products

Overall, 85% and 66% of pregnant and breastfeeding women reported having used at least 1 medicinal product (e.g., medication, folic acid, multivitamins, iron-containing preparation, omega-3 fatty acid or other product) in the 3 months prior to the survey. Specifically, at least 1 medication was used by 59% of the pregnant women (UK: 79%; IE: 67%; NL: 59%; NO: 56%; CH: 51%) (mean number of medications: 1.9; min-max: 1–15) and 56% of the breastfeeding women (UK: 68%; IE: 61%; NO: 57%; NL: 55%; CH: 52%) (mean number of medications: 2.0; min-max: 1–11). Daily use of medications was reported by 34% of pregnant women (mean number of medications: 1.5; min-max: 1–11) and occasional use by 42% (mean number of medications: 1.4; min-max: 1–10). For breastfeeding women, the prevalence of daily and occasional medication use was 29% (mean number of medications: 1.4; min-max: 1–7) and 44% (mean number of medications: 1.7; min-max: 1–8), respectively. Percentages of medication use during pregnancy and breastfeeding and according to women’s characteristics are presented in Table 1.

Multivitamins were the most commonly used other health products in pregnancy and breastfeeding. In total, 43% of pregnant and 14% of breastfeeding women reported having used multivitamins in the last 3 months. Pregnant women also commonly used folic acid (29%), iron-containing preparations (20%) and omega-3 fatty acids (13%). In contrast, few breastfeeding women indicated having used iron-containing preparations (9%), omega-3 fatty acids (6%) and folic acid (1%) in the preceding 3 months. Finally, 24% of pregnant women reported the use of any other product, while this was the case for 12% of breastfeeding women. A detailed overview of country-specific prevalences is included in the Supplementary Materials (see Table S1).

### 3.3. Types of Medication Used

The most prevalent ATC level 1 categories in pregnancy were the nervous system (30%), the respiratory system (20%), the alimentary tract/metabolism (16%), blood and blood-forming organs (6%) and systemic hormonal preparations (5%). The most prevalent categories during breastfeeding were the nervous system (37%), the respiratory system (15%), the musculo-skeletal system (14%), the genito-urinary system and sex hormones (7%), alimentary tract/metabolism (6%) and anti-infectives for systemic use (6%) (see Table 2). A detailed overview of the prevalences of ATC categories during pregnancy and breastfeeding and according to country is included in the Supplementary Materials (see Tables S2 and S3).

On ATC level 2, the most frequently reported categories during pregnancy were analgesics (28%), systemic antihistamines (15%), drugs for acid related disorders (11%), antithrombotic agents (6%) and thyroid therapy (5%). The most prevalent categories during breastfeeding were analgesics (35%), anti-inflammatory and antirheumatic products (14%), systemic antihistamines (11%), sex hormones and modulators of genital system (5%) and antibacterials for systemic use (5%) (see Supplementary Materials Table S4).

With regard to specific types of medications, the most commonly used during pregnancy were paracetamol (28%), antacids (10%), cetirizine (5%), low dose aspirin (5%), levothyroxine (4%) and omeprazole (4%). During breastfeeding, the most frequently used medications were paracetamol (34%), ibuprofen (12%), hormonal contraceptives for systemic use (5%), cetirizine (5%) and levothyroxine (4%). Daily intake of antidepressants was reported by 2% of pregnant and breastfeeding women in this cohort.

### 3.4. Factors Associated to Medication Use during Pregnancy and Breastfeeding

Pregnant women with a pre-existing chronic condition, living in the UK, older than 30 years, having been tested for SARS-CoV-2 and professionally active in healthcare had a higher likelihood of medication use than their counterparts. In contrast, women living in Switzerland and Norway were less likely to report medication use in pregnancy (see Table 3).

Moreover, breastfeeding women with a pre-existing chronic condition, having been tested for SARS-CoV-2, being in the first six months postpartum and professionally active in healthcare had a higher likelihood of medication use than their counterparts. In contrast, breastfeeding women living in Switzerland, The Netherlands and Norway were less likely to report medication use (see Table 3).

Finally, pregnant women who had a positive test result for SARS-CoV-2 were not more likely to use a medication compared to women without a positive test result (cOR 0.84; 95% CI: 0.33–2.16). A similar finding was observed for breastfeeding women (cOR 0.75; 95% CI: 0.36–1.58).

### 3.5. Attitudes towards Medication Use

Overall, 21% of women indicated being more likely to take medications during pregnancy due to the pandemic (CH: 32%; UK: 27%; NO: 26%; IE: 20%; NL: 9%). Of all breastfeeding women, 15% (strongly) agreed that they were more likely to use medications during breastfeeding as a result of the COVID-19 pandemic (NO: 24%; IE: 19%; UK: 18%; CH: 12%; NL: 4%). In addition, 22% of women reported having started the use of a medication during pregnancy on their own initiative without advice from an HCP (UK: 39%; NO: 27%; NL: 22%; IE: 19%; CH: 10%). This was the case for 16% of the women regarding the initiation of medication use during breastfeeding (NL: 21%; NO: 18%; UK: 17%; IE: 10%; CH: 10%).

## 4. Discussion

### 4.1. Main Findings

This multinational study aimed to investigate the prevalence and type of medications used by pregnant and breastfeeding women living in Europe during the first wave of the COVID-19 pandemic. To our knowledge, this is the first study examining, on a large scale, the extent and type of medications used in a perinatal cohort during this period. Overall, approximately 60% of pregnant and breastfeeding women reported the use of at least 1 medication in the 3 months preceding the survey. While medications were used daily by 1/3 of all of the participants, more than 40% reported occasional use. As women were only asked to indicate all of the products they used in the last three months, the observed prevalence rates are likely to be an underestimation of the prevalence during the overall perinatal period. Nevertheless, the findings underline that perinatal medication use was also very common during the first pandemic wave and are generally in line with prevalence estimates reported prior to the pandemic (ranging between 52 to 96% of pregnant women using a medication in the last 7 days or during their entire pregnancy, respectively, and between 50–89% of women during breastfeeding) [10,11,15,16,30,31]. Some differences across countries (and regions) in rates and types of medications used were observed to some extent, as shown earlier [10,11]. The most prevalent categories and types of medications were nervous system (paracetamol; analgesic), respiratory system (cetirizine; antihistamine), alimentary tract/metabolism (antacids; heartburn medicines) and musculo-skeletal system (ibuprofen; analgesic), which was generally quite similar to the findings obtained during pre-pandemic studies in the perinatal population [10,15,31].

However, some additional comments could be made with regard to the use of two specific groups of medications. First, the rate of antibacterial use during pregnancy and postpartum was lower during the pandemic than previously reported (3% vs. 15% and 6% vs. 8%, respectively). [10,15]. A decreased rate of antibiotic consumption was also recently observed in the primary care population [19,21,32], and might be explained by the widespread infection control measures taken in the fight against SARS-CoV-2. It is also likely that the limited (physical) access to perinatal healthcare services during the pandemic led to a decreased likelihood of getting a prescription for antibiotics [3,4]. Although a reduced consumption of antibiotics is an important global objective to prevent antibiotic resistance, the timely diagnosis and treatment of infectious diseases could have been hindered in some cases during the lockdown. This delay could be potentially harmful in case of undiagnosed infections associated with adverse pregnancy outcomes such as asymptomatic bacteriuria [33]. This finding and related considerations requires further investigation but should meanwhile encourage HCPs to remain vigilant in clinical practice.

Second, the observed prevalence of antidepressant use in our cohort (2%) was similar to pre-pandemic estimates, despite the high prevalence of depressive symptoms in this cohort during the study period [10,25]. This finding is in contrast with recent studies in non-perinatal samples showing increased exposure to antidepressants and psychotropics during the pandemic [22,23,24]. The low rate of antidepressant and psychotropic use in our cohort compared to the high prevalence of mental distress might have been caused by limited or delayed diagnosis of depression or anxiety by HCPs. This may add to the growing body of evidence of reduced access to perinatal healthcare during the pandemic [3,4,5]. The likelihood of pandemic-related shifts in utilization patterns of psychotropic medications in pregnancy and the postpartum period should be further studied in other cohorts. In any case, HCPs should pay close attention to perinatal mental health given its high prevalence during the pandemic and its long-term importance in safeguarding infant mental health [8,25].

Moreover, factors associated with medication use in pregnancy and breastfeeding were having a chronic illness, country of residence, maternal age, SARS-CoV-2 testing, professional status and time since delivery. The likelihood of medication use in pregnancy increased with higher age, as shown earlier [10,11,31,34]. Although women working in healthcare were more likely to use medications compared to women not employed in healthcare, the absolute difference between both groups was small (63% vs. 55–57%). This finding aligns with a previous study showing that pregnant women working as HCPs were more likely to use short-term and over-the-counter (OTC) medications [10]. Furthermore, it is not surprising that medication use was higher among women tested for SARS-CoV-2 in this study covering the first pandemic wave. Due to the limited test capacity, patients tested in the early phases of the pandemic were more likely to have severe symptoms, and thus use medications, compared to later stages of the pandemic where testing became more easily accessible. In addition, the observation that about 20% of women reported being more likely to take medications as a result of the pandemic deserves further attention. This is particularly important given our observation of the self-initiation of medications, some of them having potential detrimental effects on the developing fetus (e.g., ibuprofen use after 20 weeks) [35]. Future studies should examine which medications women are more likely to use during a pandemic, and which safety issues ensued as a result.

### 4.2. Strengths and Limitations

This study has several strengths. First, a large perinatal sample of women living in geographically dispersed countries across Europe was obtained (>8000 participants). Uniform data collection and analysis was applied to measure medication use, allowing the comparison of results between countries. Following uniform medication coding rules, data were categorized and coded by national coordinators who are familiar with country-specific medicinal products and brand names, thereby increasing the validity of the findings. Providing anonymity for the respondents may have stimulated women to disclose all of the products they used in the previous months, thereby enhancing the collection of complete data.

Some limitations can also be addressed. First, a self-reporting web survey distributed via social media was used. This approach may have led to selection bias limiting the generalizability of the findings. Compared to national birthing statistics, respondents were more educated and more likely to be professionally active (in healthcare), in a relationship and first-time mother, and were often in their third gestational trimester. As low education might be associated with a higher use of medications according to prior studies [10,36], the prevalence estimates observed in our cohort might be an underestimation of the true rates during the first wave of the pandemic. On the other hand, this might be counteracted by the higher proportion of women working in healthcare who might be more likely to use medications [10]. The overrepresentation of women in their third trimester might explain the low rate of (multi)vitamin use in the preceding three months and the high proportion of antacid users. Some degree of recall bias due to the applied survey question to report all medicinal products used in the last three months and any seasonal influences on utilization patterns could also not be excluded. Second, the cross-sectional design and the lack of controls prevented us from drawing sound conclusions on the actual impact of the pandemic on medication use. As breastfeeding women who delivered recently were also included in the study sample, it cannot be excluded that some of the medications recorded by these women correspond to exposure during pregnancy. Thirdly, only few respondents had a positive SARS-CoV-2 test, while the timing of testing was unknown. Hence, this study does not provide insight into the extent and type of medications used by pregnant and breastfeeding women with confirmed COVID-19. In contrast to the other countries, only few data were obtained in the UK. Finally, no data were collected on the indication for which medications were used, the level of medication adherence, which medications women started on their own initiative, nor whether women stopped using some medications due to media reports on beneficial or adverse effects on COVID-19 outcomes or after advice from a HCP [18,37]. The results should be interpreted bearing in mind the strengths and limitations of the study. Future studies should also further explore the impact of women’s attitudes on perinatal medication utilization patterns.

## 5. Conclusions

In this study, we found that medication use during pregnancy and breastfeeding was highly prevalent during the first wave of the pandemic, with approximately 60% of pregnant and breastfeeding women having used at least 1 medication in the 3 months preceding the survey. The most prevalent ATC categories were the nervous system, the respiratory system, the alimentary tract/metabolism, and the musculo-skeletal system. The most frequently used medications were paracetamol, ibuprofen, antacids, and cetirizine. In general, a similar pattern of medication use was observed compared to prior to COVID-19, including for antidepressants. However, the rate of antibacterial use during pregnancy and postpartum was lower than previously reported. Future studies should further investigate the extent of pandemic-related shifts in the use of specific medication groups. Nevertheless, the high prevalence of perinatal medication use during the COVID-19 pandemic clearly underlines the importance of maintaining counseling efforts and guidance on perinatal medication use, even in times of disrupted healthcare services and/or limited resources.

## Figures and Tables

**Table 1:** Characteristics of participants according to any medication use in the preceding 3 months (*n* = 8378).

	Pregnant Women (*n* = 3 666)	Pregnant Women Using Medication in Pregnancy (*n* = 1958)	Breastfeeding Women (*n* = 4712)	Women Using Medication While Breastfeeding (*n* = 2413)
	*n* (%)	*n* (%)	*n* (%)	*n* (%)
	**Sociodemographic characteristics**			
**Country**				
Norway The Netherlands Ireland Switzerland United Kingdom	1344 (36.7) 1073 (29.3) 619 (16.9) 515 (14.0) 115 (3.1)	751 (55.9) 525 (59.3) 375 (66.5) 223 (50.9) 84 (78.5)	1485 (31.5) 1314 (27.9) 799 (17.0) 1035 (22.0) 79 (1.7)	852 (57.4) 614 (55.1) 442 (61.0) 456 (51.7) 49 (68.1)
**Maternal age (years)**				
18–25 26–30 31–35 36–40 >40	274 (8.5) 1069 (33.1) 1290 (40.0) 532 (16.5) 64 (2.0)	149 (54.4) 601 (56.2) 776 (60.2) 346 (65.0) 47 (73.4)	164 (4.0) 1062 (25.8) 1864 (45.4) 858 (20.9) 161 (3.9)	88 (53.7) 596 (56.1) 1073 (57.6) 511 (59.6) 92 (57.1)
**Relationship status**				
Partner No partner	3195 (98.4) 51 (1.6)	1897 (59.4) 30 (58.8)	4076 (98.5) 62 (1.5)	2342 (57.5) 33 (53.2)
**Professional status**				
Professionally active, not in healthcare Professionally active, in healthcare Not professionally active	1890 (59.4) 991 (31.1) 302 (9.5)	1075 (56.9) 625 (63.1) 187 (61.9)	2412 (59.2) 1204 (29.5) 461 (11.3)	1323 (54.9) 756 (62.8) 259 (56.2)
**Highest education level**				
Low Medium High	176 (5.6) 808 (25.6) 2168 (68.8)	109 (61.9) 467 (57.8) 1307 (60.3)	292 (7.3) 984 (24.7) 2713 (68.0)	151 (51.7) 537 (54.6) 1598 (58.9)
**Smoking in pregnancy/breastfeeding**	85 (2.6)	52 (61.2)	140 (3.4)	72 (51.4)
**Health and reproductive characteristics**				
**SARS-CoV-2**				
Tested ^a^ Tested positive	325 (8.9) 21 (0.6)	208 (69.6) 14 (66.7)	514 (10.9) 33 (0.7)	327 (69.3) 20 (62.5)
**Chronic illness ^b^**	623 (19.2)	509 (81.7)	665 (16.1)	538 (80.9)
**Depressive symptoms** (EDS ≥ 13)	501 (15.0)	323 (64.5)	622 (14.5)	383 (61.6)
**Gestational trimester ^c^**				
First trimester (0–12 weeks) Second trimester (13–24 weeks) Third trimester (25–40 weeks)	280 (7.8) 916 (25.6) 2388 (66.6)	138 (57.3) 517 (61.4) 1265 (57.8)	N/A N/A N/A	N/A N/A N/A
**Parity**				
Nulliparous Multiparous	1964 (53.7) 1692 (46.3)	1031 (57.7) 920 (59.6)	N/A N/A	N/A N/A
**Planned pregnancy**				
Yes No No, but it was not unexpected	2938 (80.1) 246 (6.7) 482 (13.1)	1570 (58.8) 130 (58.0) 258 (58.0)	N/A N/A N/A	N/A N/A N/A
**Current breastfeeding duration**				
≤6 weeks Between 6 weeks–6 months >6 months	N/A N/A N/A	N/A N/A N/A	708 (15.0) 2301 (48.8) 1703 (36.1)	375 (59.3) 1238 (58.5) 800 (52.3)
**Previous breastfeeding experience**	N/A	N/A	2639 (56.0)	1333 (55.7)

Results are expressed as absolute numbers (%). Numbers may not add up due to missing values. N/A = question was not applicable. EDS = Edinburgh Depression Scale [29]. ^a^ Refers to having been tested for SARS-CoV-2 ever in life since the start of the pandemic. ^b^ A chronic illness refers to conditions that already existed prior to pregnancy. ^c^ Gestational trimester at study enrollment.

**Table 2:** Any, daily and occasional medication use on 1st ATC level among pregnant and breastfeeding women.

	Pregnant Women (*n* = 3339)			Breastfeeding Women (*n* = 4278)		
Medication Category (ATC 1st Level)	Any Use *n* (%)	Daily Use *n* (%)	Occasional Use *n* (%)	Any Use *n* (%)	Daily Use *n* (%)	Occasional Use *n* (%)
Any medication use/category	1958 (58.6)	1128 (33.8)	1399 (41.9)	2413 (56.4)	1249 (29.2)	1866 (43.6)
Nervous system (N)	996 (29.8)	138 (4.1)	909 (27.2)	1565 (36.6)	241 (5.6)	1384 (32.4)
Respiratory system (R)	671 (20.1)	417 (12.5)	324 (9.7)	642 (15.0)	392 (9.2)	324 (7.6)
Alimentary tract and metabolism (A)	547 (16.4)	313 (9.4)	286 (8.6)	271 (6.3)	161 (3.8)	126 (2.9)
Blood and blood-forming organs (B)	208 (6.2)	206 (6.2)	4 (0.1)	62 (1.4)	51 (1.2)	12 (0.3)
Systemic hormonal preparations (H)	163 (4.9)	153 (4.6)	9 (0.3)	220 (5.1)	181 (4.2)	37 (0.9)
Antiinfectives for systemic use (J)	103 (3.1)	39 (1.2)	70 (2.1)	241 (5.6)	62 (1.4)	187 (4.4)
Genito urinary system & sex hormones (G)	91 (2.7)	53 (1.6)	41 (1.2)	282 (6.6)	242 (5.7)	47 (1.1)
Cardiovascular system (C)	85 (2.5)	60 (1.8)	26 (0.8)	114 (2.7)	77 (1.8)	43 (1.0)
Musculoskeletal system (M)	27 (0.8)	4 (0.1)	24 (0.7)	613 (14.3)	56 (1.3)	563 (13.2)

Results are expressed as absolute numbers (%). Only ATC categories for which at least one of the two percentages for any medication use during pregnancy or breastfeeding in the preceding three months was higher than two percent are shown.

**Table 3:** Factors associated to medication use among pregnant and breastfeeding women.

	Pregnant Women		Breastfeeding Women	
	cOR * (95% CI)	aOR ** (95% CI)	cOR * (95% CI)	aOR *** (95% CI)
**Country**				
Ireland Norway Switzerland The Netherlands UK	Ref 0.64 (0.52–0.78) 0.52 (0.41–0.68) 0.73 (0.59–0.91) 1.84 (1.12–3.01)	Ref **0.61 (0.48–0.76)** **0.58 (0.44–0.77)** 0.84 (0.66–1.07) **1.98 (1.14–3.46)**	Ref 0.86 (0.72–1.03) 0.68 (0.56–0.83) 0.78 (0.65–0.95) 1.36 (0.81–2.28)	Ref **0.80 (0.65–0.98)** **0.74 (0.59–0.93)** **0.77 (0.62–0.95)** 1.09 (0.62–1.90)
**Maternal age (years)**				/
18–25 26–30 31–35 36–40 >40	Ref 1.08 (0.83–1.41) 1.27 (0.97–1.65) 1.56 (1.16–2.10) 2.32 (1.27–4.24)	Ref 1.18 (0.89–1.58) **1.40 (1.05–1.86)** **1.61 (1.17–2.23)** **2.58 (1.36–4.92)**	Ref 1.11 (0.79–1.54) 1.17 (0.85–1.61) 1.27 (0.91–1.78) 1.15 (0.74–1.78)	/ / / / /
**Professional status**				
Active, but not in healthcare Active in healthcare Not professionally active	Ref 1.30 (1.11–1.52) 1.23 (0.96–1.58)	Ref **1.23 (1.04–1.46)** 1.01 (0.77–1.33)	Ref 1.39 (1.21–1.60) 1.06 (0.86–1.29)	Ref **1.36 (1.17–1.59)** 1.00 (0.80–1.24)
**Tested for SARS-CoV-2**	1.69 (1.30–2.18)	**1.46 (1.10–1.92)**	1.86 (1.51–2.28)	**1.81 (1.43–2.28)**
**Depressive symptoms (EDS ≥ 13)**	1.34 (1.10–1.63)	1.20 (0.96–1.49)	1.28 (1.08–1.53)	1.08 (0.89–1.33)
**Having a chronic illness**	3.79 (3.05–4.71)	**4.08 (3.25–5.13)**	3.77 (3.08–4.63)	**3.74 (3.00–4.65)**
**Breastfeeding duration**				
≤6 weeks Between 6 weeks–6 months >6 months	N/A N/A N/A	N/A N/A N/A	1.35 (1.11–1.64) 1.30 (1.13–1.49) Ref	**1.35 (1.10–1.66)** **1.34 (1.16–1.55)** Ref

N/A = not available; CI = confidence interval; * cOR = crude odds ratio; ** aOR = adjusted odds ratio, adjusted for country, maternal age, professional status, tested for SARS-CoV-2, EDS ≥ 13, having a chronic illness. *** aOR, adjusted for country, professional status, tested for SARS-CoV-2, EDS ≥ 13, having a chronic illness, and breastfeeding duration. The bold numbers indicate aORs where the 95% CI does not include 1.

## Data Availability

The collected data are presented in the manuscript and in the Supplementary Materials.

## Supplementary Materials

The following are available online at https://www.mdpi.com/article/10.3390/ijerph19031389/s1. Figure S1: English version of the questionnaire relevant to this manuscript. Table S1: Prevalence of the use of other health products during pregnancy and breastfeeding according to country; Table S2: Medication use during pregnancy on 1st ATC level according to country. Table S3: Medication use during breastfeeding on 1st ATC level according to country. Table S4: Daily, occasional and total medication use on 1st and 2nd ATC level in the last three months among pregnant and breastfeeding women.
