# Supplementary material for: Self-Reported Medication Use among Pregnant and Breastfeeding Women during the COVID-19 Pandemic: A Cross-Sectional Study in Five European Countries
Source: Int J Environ Res Public Health. 2022 Jan 26;19(3):1389. doi: 10.3390/ijerph19031389 (PMC8835534; doi:10.3390/ijerph19031389)
Supplement: Supplementary file 1 [file ijerph-19-01389-s001.zip › ijerph-1547857-supplementary.pdf]

Figure S1: English version of the questionnaire relevant to this manuscript.

YOUR PREGNANCY

1. How many weeks pregnant are you right now? (open answer)

2. Was the pregnancy planned?
   a. Yes
   b. No
   c. No, but it was not unexpected

3. Have you been pregnant before?
   a. Yes
   b. No

4. If yes: How many children do you already have (not including the current pregnancy) (write your answer down in numbers)?

CORONAVIRUS SYMPTOMS

5. Did you receive a test to determine coronavirus infection?
   a. Yes
   b. No

6. If yes: What was the result? If you received multiple tests, please indicate ‘positive’ if at least one of the test results was positive
   a. Positive (infected with the coronavirus)
   b. Negative (not infected with the coronavirus)
   c. I don’t know (yet)

CORONAVIRUS AND PERINATAL EXPERIENCES

Please indicate to which extent you agree with the following statement:

7. Due to the coronavirus, I am more likely to take medication during pregnancy.
    a. Strongly agree
    b. Agree
    c. Disagree
    d. Strongly disagree

CORONAVIRUS AND YOUR WELLBEING

We would like to know how you are feeling.

Please indicate the answer which comes closest to how you have felt in the past 7 days, not just how you feel today.

In the past 7 days:

8. I have been able to laugh and see the funny side of things
    a. As much as I always could
    b. Not quite so much now
    c. Definitely not so much now
    d. Not at all

9. I have looked forward with enjoyment to things.
    a. As much as I ever did
    b. Rather less than I used to
    c. Definitely less than I used to
    d. Hardly at all

10. I have blamed myself unnecessarily when things went wrong.
   a. Yes, most of the time
   b. Yes, some of the time
   c. Not very often
   d. No, never

11. I have been anxious or worried for no good reason.
   a. No, not at all
   b. Hardly ever
   c. Yes, sometimes
   d. Yes, very often

12. I have felt scared or panicky for no very good reason.
   a. Yes, quite a lot
   b. Yes, sometimes
   c. No, not much
   d. No, not at all

13. Things have been getting on top of me.
   a. Yes, most of the time I haven't been able to cope at all
   b. Yes, sometimes I haven't been coping as well as usual
   c. No, most of the time I have coped quite well
   d. No, I have been coping as well as ever

14. I have been so unhappy that I have had difficulty sleeping.
    a. Yes, most of the time
    b. Yes, sometimes
    c. Not very often
    d. No, not at all

15. I have felt sad or miserable.
    a. Yes, most of the time
    b. Yes, quite often
    c. Not very often
    d. No, not at all

16. I have been so unhappy that I have been crying.
    a. Yes, most of the time
    b. Yes, quite often
    c. Only occasionally
    d. No, never

17. The thought of harming myself has occurred to me.
    a. Yes, quite often
    b. Sometimes
    c. Hardly ever
    d. Never

USE OF MEDICINES

18. Did you use any medication(s) in the past 3 months?

    Please include all prescription and non-prescription medicines, folic acid, and herbal medicines.

    a. Yes
    b. No

19. If yes: Please write down the name(s) of the medication(s) you are currently using on a daily basis. Please write down the symbol ";" in between every medication (E.g.: cetirizine;ranitidine;…). You may choose to write down the brand name or the generic name.

20. If yes, please write down the name(s) of the medication(s) you have used occasionally. Please write down the symbol ";" in between every medication. (E.g.: paracetamol; salbutamol inhaler;…). You may choose to write down the brand name or the generic name.

21. Did you start to use any medication(s) during your pregnancy on own initiative without advice of a healthcare professional?

    a. Yes
    b. No

PERSONAL BACKGROUND

22. Year of birth?

23. Do you currently have a partner?

    a. Yes
    b. No

24. Have you smoked in the past 4 weeks?

a. Yes
b. No

25. Do you have any chronic illnesses (these are conditions that already existed before your pregnancy)?

a. Yes
b. No

26. If yes: which chronic illness(es) do you have?

a. Asthma
b. Allergy
c. Cardiovascular disease (including high blood pressure, high cholesterol, heart disease,..)
d. Depression
e. Diabetes
f. Epilepsy
g. Hypothyroidism (underactive thyroid)
h. Rheumatic diseases (including rheumatoid arthritis, psoriatic arthritis,…)
i. Other: ………………………………..

27. What is your highest degree?

a. Primary education
b. Professional secondary education
c. Technical secondary education
d. Artistic secondary education
e. General secondary education

f. Professional bachelor
g. Academic bachelor
h. Master
i. PhD
j. Other: ……….

28. What is your current professional status? (if you are currently not working because of pregnancy complications or pregnancy leave, please fill in the professional status you had before)

a. Employee
    a. Are/were you working in healthcare?
b. Self-employed
    a. Are/were you working in healthcare?
c. Civil service employee
    a. Are/were you working in healthcare?
d. Student
    a. Are you following an education in healthcare?
e. Housewife
f. Jobseeker/unemployed
g. Incapacitated/disabled
h. Other: ………

CLOSING AND THANKS

29. The coronavirus affected many people in many different ways, both negatively and/or positively.
Please share any of your personal experiences or anything that has changed in your life due to the coronavirus. (Optional, open field)

CORONAVIRUS AND BREASTFEEDING

1. Are you currently breastfeeding?
   a. Yes
   b. No

2. Please indicate the age of the baby that is currently breastfed / that was breastfed in the last 3 months.
   a. ≤ 6 weeks
   b. Between 6 weeks and 6 months
   c. > 6 months

3. Did you ever breastfeed before the coronavirus outbreak?
   a. Yes
   b. No

CORONAVIRUS SYMPTOMS

4. Did you receive a test to determine coronavirus infection?
   a. Yes
   b. No

5. If yes: What was the result of the test? If you received multiple tests, please indicate 'positive' if at least one of the test results was positive
   a. Positive (infected with the coronavirus)
   b. Negative (not infected with the coronavirus)
   c. I don't know (yet)

CORONAVIRUS AND PERINATAL EXPERIENCES

Please indicate to which extent you agree with the following statement:

6. Due to the coronavirus, I am more likely to take medication during breastfeeding.
    a. Strongly agree
    b. Agree
    c. Disagree
    d. Strongly disagree

CORONAVIRUS AND YOUR WELLBEING

We would like to know how you are feeling.

Please indicate the answer which comes closest to how you have felt in the past 7 days, not just how you feel today.

In the past 7 days:

7. I have been able to laugh and see the funny side of things
    a. As much as I always could
    b. Not quite so much now
    c. Definitely not so much now
    d. Not at all

8. I have looked forward with enjoyment to things.
    a. As much as I ever did
    b. Rather less than I used to
    c. Definitely less than I used to
    d. Hardly at all

9. I have blamed myself unnecessarily when things went wrong.
    a. Yes, most of the time
    b. Yes, some of the time
    c. Not very often
    d. No, never

10. I have been anxious or worried for no good reason.
    a. No, not at all
    b. Hardly ever
    c. Yes, sometimes
    d. Yes, very often

11. I have felt scared or panicky for no very good reason.
    a. Yes, quite a lot
    b. Yes, sometimes
    c. No, not much
    d. No, not at all

12. Things have been getting on top of me.
    a. Yes, most of the time I haven't been able to cope at all
    b. Yes, sometimes I haven't been coping as well as usual
    c. No, most of the time I have coped quite well
    d. No, I have been coping as well as ever

13. I have been so unhappy that I have had difficulty sleeping.
    a. Yes, most of the time
    b. Yes, sometimes
    c. Not very often
    d. No, not at all

14. I have felt sad or miserable.
    a. Yes, most of the time
    b. Yes, quite often
    c. Not very often
    d. No, not at all

15. I have been so unhappy that I have been crying.
    a. Yes, most of the time
    b. Yes, quite often
    c. Only occasionally
    d. No, never

16. The thought of harming myself has occurred to me.
    a. Yes, quite often
    b. Sometimes
    c. Hardly ever
    d. Never

USE OF MEDICINES

17. Did you use any medication(s) in the past 3 months? *Please include all prescription, non-prescription, and herbal medicines.*
    a. Yes
    b. No

18. If yes: Please write down the name(s) of the medication(s) you are currently using on a daily basis. Please write down the symbol ";" in between every medication (E.g.: cetirizine;ranitidine;…). You may choose to write down the brand name or the generic name.

19. If yes, please write down the name(s) of the medication(s) you have used occasionally. Please write down the symbol ";" in between every medication. (E.g.: paracetamol;salbutamol inhaler;…). You may choose to write down the brand name or the generic name.

20. Did you start to use any medication(s) during breastfeeding on own initiative without advice of a healthcare professional?
    a. Yes
    b. No

PERSONAL BACKGROUND

21. Year of birth?

22. Do you currently have a partner?
    a. Yes
    b. No

23. Have you smoked in the past 4 weeks?

c. Yes

d. No

24. Do you have any chronic illnesses? (these are conditions that already existed before your pregnancy)?

a. Yes

b. No

25. If yes: what chronic illness do you have?

a. Asthma

b. Allergy

c. Cardiovascular disease (including high blood pressure, high cholesterol, heart disease)

d. Depression

e. Diabetes

f. Epilepsy

g. Hypothyroidism (underactive thyroid)

h. Rheumatic diseases (including rheumatoid arthritis, psoriatic arthritis)

i. Other: ………………………………..

26. What is your highest degree?

a. Primary education

b. Professional secondary education

c. Technical secondary education

d. Artistic secondary education

e. General secondary education

f. Professional bachelor
g. Academic bachelor
h. Master
i. PhD
j. Other: ……….

27. What is your current professional status? (if you are currently not working because of maternity or parental leave, please fill in the professional status you had before)

a. Employee
    1. Are/were you working in healthcare?
b. Self-employed
    1. Are/were you working in healthcare?
c. Civil service employee
    1. Are/were you working in healthcare?
d. Student
    1. Are you following an education in healthcare?
e. Housewife
f. Jobseeker/unemployed
g. Incapacitated/disabled
h. Other: ………

CLOSING AND THANKS

28. The coronavirus affected many people in many different ways, both negatively and/or positively.

Please share any of your personal experiences or anything that has changed in your life due to the coronavirus. (Optional, open field)

Table S1: Prevalence of the use of other health products during pregnancy & breastfeeding according to country.

	Pregnant women						Breastfeeding women					
	**Overall** (N=3339) *N (%)*	**Ireland** (N=564) *N (%)*	**Norway** (N=1344) *N (%)*	**Switzerland** (N=438) *N (%)*	**Netherlands** (N=886) *N (%)*	**UK** (N=107) *N (%)*	**Overall** (N=4278) *N (%)*	**Ireland** (N=724) *N (%)*	**Norway** (N=1485) *N (%)*	**Switzerland** (N=882) *N (%)*	**Netherlands** (N=1115) *N (%)*	**UK** (N=72) *N (%)*
**Multivitamins**	1420 (42.5)	296 (52.5)	546 (40.6)	234 (53.4)	296 (33.4)	48 (44.9)	582 (13.6)	64 (8.8)	342 (23.0)	136 (15.4)	37 (3.3)	3 (4.2)
**Folic acid**	983 (29.4)	160 (28.4)	414 (30.8)	95 (21.7)	277 (31.3)	37 (34.6)	55 (1.3)	8 (1.1)	31 (2.1)	8 (0.9)	6 (0.5)	2 (2.8)
**Iron-containing preparations**	665 (19.6)	113 (20.0)	364 (27.1)	97 (22.1)	65 (7.3)	16 (15.0)	379 (8.9)	73 (10.1)	203 (13.7)	48 (5.4)	53 (4.8)	2 (2.8)
**Omega-3 containing products**	422 (12.6)	36 (6.4)	335 (24.9)	30 (6.8)	17 (1.9)	4 (3.7)	269 (6.3)	3 (0.4)	251 (16.9)	11 (1.2)	4 (0.4)	0 (0.0)
**Other products[1]**	788 (23.6)	86 (15.2)	327 (24.3)	179 (40.9)	166 (18.7)	30 (28.0)	494 (11.5)	50 (6.9)	237 (16.0)	121 (13.7)	82 (7.4)	4 (5.6)

Results are expressed as absolute numbers (%). [1] The group 'other products' consists of all types of health products that could not be classified elsewhere, including but not limited to pre- and probiotics, herbal remedies and homeopathic products.

Table S2: Medication use during pregnancy on 1st ATC level according to country.

Medication category (ATC 1st level)	Any use (N=3339) *N (%)*	Ireland (N=564) *N (%)*	Norway (N=1344) *N (%)*	Switzerland (N=438) *N (%)*	Netherlands (N=886) *N (%)*	UK (N=107) *N (%)*
Nervous system (N)	996 (29.8)	244 (43.3)	292 (21.7)	113 (25.8)	289 (32.6)	58 (54.2)
Respiratory system (R)	671 (20.1)	82 (14.5)	368 (27.4)	46 (10.5)	151 (17.0)	24 (22.4)
Alimentary tract and metabolism (A)	547 (16.4)	102 (18.1)	179 (13.3)	64 (14.6)	172 (19.4)	30 (28.0)
Blood and blood-forming organs (B)	208 (6.2)	63 (11.2)	58 (4.3)	21 (4.8)	53 (6.0)	13 (12.1)
Systemic hormonal preparations (H)	163 (4.9)	43 (7.6)	64 (4.8)	33 (7.5)	21 (2.4)	<3 (<2.8)
Anti-infectives for systemic use (J)	103 (3.1)	24 (4.3)	34 (2.5)	12 (2.7)	28 (3.2)	5 (4.7)
Genito urinary system and sex hormones (G)	91 (2.7)	16 (2.8)	40 (3.0)	17 (3.9)	13 (1.5)	5 (4.7)
Cardiovascular system (C)	85 (2.5)	25 (4.4)	24 (1.8)	11 (2.5)	19 (2.1)	6 (5.6)
Musculoskeletal system (M)	27 (0.8)	<3 (<0.5)	7 (0.5)	8 (1.8)	7 (0.8)	4 (3.7)

Results are expressed as absolute numbers (%).

Table S3: Medication use during breastfeeding on 1st ATC level according to country.

Medication category (ATC 1st level)	Any use (N=4278) *N (%)*	Ireland (N=724) *N (%)*	Norway (N=1485) *N (%)*	Switzerland (N=882) *N (%)*	the Netherlands (N=1115) *N (%)*	UK (N=72) *N (%)*
Nervous system (N)	1565 (36.6)	328 (45.3)	481 (32.4)	332 (37.6)	394 (35.3)	30 (41.7)
Respiratory system (R)	642 (15.0)	86 (11.9)	342 (23.0)	66 (7.5)	135 (12.1)	13 (18.1)
Musculoskeletal system (M)	613 (14.3)	158 (21.8)	221 (14.9)	143 (16.2)	79 (7.1)	12 (16.7)
Genito urinary system and sex hormones (G)	282 (6.6)	46 (6.4)	123 (8.3)	48 (5.4)	59 (5.3)	6 (8.3)
Alimentary tract and metabolism (A)	271 (6.3)	61 (8.4)	93 (6.3)	31 (3.5)	79 (7.1)	7 (9.7)
Anti-infectives for systemic use (J)	241 (5.6)	68 (9.4)	82 (5.5)	25 (2.8)	58 (5.2)	8 (11.1)
Systemic hormonal preparations (H)	220 (5.1)	45 (6.2)	80 (5.4)	41 (4.6)	49 (4.4)	5 (6.9)
Cardiovascular system (C)	114 (2.7)	20 (2.8)	51 (3.4)	16 (1.8)	23 (2.1)	4 (5.6)
Blood and blood-forming organs (B)	62 (1.4)	13 (1.8)	25 (1.7)	9 (1.0)	14 (1.3)	<3 (<4.2)

Results are expressed as absolute numbers (%).

Table S4: Daily, occasional, and total medication use on 1st and 2nd ATC level in the last three months among pregnant and breastfeeding women.

**Anatomical Therapeutic Chemical (ATC) classification index 1st and 2nd levels***		Pregnant women N = 3339			Breastfeeding women N = 4278		
		Daily use N (%)	Occasional use N (%)	Total N (%)	Daily use N (%)	Occasional use N (%)	Total N (%)
**A**	**Alimentary tract and metabolism**	**313 (9.4)**	**286 (8.6)**	**547 (16.4)**	**161 (3.8)**	**126 (2.9)**	**271 (6.3)**
**A01**	Stomatological preparations	<3 (0.1)	0 (0.0)	<3 (0.1)	<3 (0.1)	<3 (0.1)	3 (0.1)
**A02**	*Drugs for acid related disorders*	184 (5.5)	202 (6.0)	367 (11.0)	70 (1.6)	67 (1.6)	127 (3.0)
**A03**	Drugs for functional gastrointestinal disorders	29 (0.9)	39 (1.2)	64 (1.9)	16 (0.4)	19 (0.4)	34 (0.8)
**A04**	Antiemetics and antinauseants	11 (0.3)	16 (0.5)	27 (0.8)	<3 (0.1)	4 (0.1)	4 (0.1)
**A05**	Bile and liver therapy	<3 (0.1)	0 (0.0)	<3 (0.1)	3 (0.1)	<3 (0.1)	4 (0.1)
**A06**	Laxatives	36 (1.1)	39 (1.2)	73 (2.2)	36 (0.8)	34 (0.8)	69 (1.6)
**A07**	Antidiarrheals	16 (0.5)	4 (0.1)	20 (0.6)	21 (0.5)	8 (0.2)	28 (0.7)
**A08**	Antiobesity preparations, excl. diet products	0 (0.0)	0 (0.0)	0 (0.0)	<3 (0.1)	0 (0.0)	<3 (0.1)
**A09**	Digestives, incl. enzymes	<3 (0.1)	0 (0.0)	<3 (0.1)	0 (0.0)	0 (0.0)	0 (0.0)
**A10**	Drugs used in diabetes	56 (1.7)	8 (0.2)	58 (1.7)	20 (0.5)	0 (0.0)	20 (0.5)
**B**	**Blood and blood-forming organs**	**206 (6.2)**	**4 (0.1)**	**208 (6.2)**	**51 (1.2)**	**12 (0.3)**	**62 (1.4)**
**B01**	*Antithrombotic agents*	205 (6.1)	4 (0.1)	207 (6.2)	49 (1.1)	11 (0.3)	59 (1.4)
**B02**	Antihemorrhagics	0 (0.0)	0 (0.0)	0 (0.0)	<3 (0.1)	<3 (0.1)	3 (0.1)
**C**	**Cardiovascular system**	**60 (1.8)**	**27 (0.8)**	**85 (2.5)**	**77 (1.8)**	**43 (1.0)**	**114 (2.7)**
**C01**	Cardiac therapy	3 (0.1)	0 (0.0)	3 (0.1)	0 (0.0)	0 (0.0)	0 (0.0)
**C02**	Antihypertensives	12 (0.4)	0 (0.0)	12 (0.4)	13 (0.3)	7 (0.2)	20 (0.5)
**C03**	Diuretics	<3 (0.1)	0 (0.0)	<3 (0.1)	<3 (0.1)	0 (0.0)	<3 (0.1)
**C05**	Vasoprotectives	<3 (0.1)	19 (0.6)	20 (0.6)	10 (0.2)	24 (0.6)	31 (0.7)
**C07**	*Beta blocking agents*	40 (1.2)	5 (0.1)	44 (1.3)	45 (1.1)	8 (0.2)	53 (1.2)
**C08**	Calcium channel blockers	6 (0.2)	<3 (0.1)	8 (0.2)	14 (0.3)	5 (0.1)	19 (0.4)

**Anatomical Therapeutic Chemical (ATC) classification index 1st and 2nd levels***		Pregnant women N = 3339			Breastfeeding women N = 4278		
		Daily use N (%)	Occasional use N (%)	Total N (%)	Daily use N (%)	Occasional use N (%)	Total N (%)
**C09**	Agents acting on the renin-angiotensin system	0 (0.0)	0 (0.0)	0 (0.0)	<3 (0.1)	0 (0.0)	<3 (0.1)
**C10**	Lipid modifying agents	<3 (0.1)	<3 (0.1)	<3 (0.1)	<3 (0.1)	0 (0.0)	<3 (0.1)
**D**	**Dermatologicals**	**11 (0.3)**	**51 (1.5)**	**60 (1.8)**	**30 (0.7)**	**68 (1.6)**	**95 (2.2)**
**D01**	*Antifungals for dermatological use*	4 (0.1)	19 (0.6)	23 (0.7)	11 (0.3)	30 (0.7)	40 (0.9)
**D02**	Emollients and protectives	0 (0.0)	5 (0.1)	5 (0.1)	<3 (0.1)	<3 (0.1)	3 (0.1)
**D03**	Preparations for treatment of wounds & ulcers	0 (0.0)	0 (0.0)	0 (0.0)	0 (0.0)	<3 (0.1)	<3 (0.1)
**D04**	Antipruritics, incl. antihistamines, anesthetics.	<3 (0.1)	<3 (0.1)	3 (0.1)	<3 (0.1)	4 (0.1)	5 (0.1)
**D05**	Antipsoriatics	0 (0.0)	<3 (0.1)	<3 (0.1)	<3 (0.1)	<3 (0.1)	<3 (0.1)
**D06**	Antibiotics and chemotherapeutics for dermatological use	<3 (0.1)	4 (0.1)	6 (0.2)	7 (0.2)	9 (0.2)	16 (0.4)
**D07**	Corticosteroids, dermatological preparations	3 (0.1)	24 (0.7)	25 (0.7)	10 (0.2)	25 (0.6)	35 (0.8)
**D08**	Antiseptics and disinfectants	0 (0.0)	<3 (0.1)	<3 (0.1)	0 (0.0)	<3 (0.1)	<3 (0.1)
**D10**	Anti-acne preparations	<3 (0.1)	<3 (0.1)	<3 (0.1)	3 (0.1)	0 (0.0)	3 (0.1)
**D11**	Other dermatological preparations	0 (0.0)	<3 (0.1)	<3 (0.1)	0 (0.0)	0 (0.0)	0 (0.0)
**G**	**Genito urinary system and sex hormones**	**53 (1.6)**	**41 (1.2)**	**91 (2.7)**	**242 (5.7)**	**47 (1.1)**	**282 (6.6)**
**G01**	Gynecological anti-infectives and antiseptics	4 (0.1)	31 (0.9)	35 (1.0)	11 (0.3)	22 (0.5)	33 (0.8)
**G02**	Other gynecologicals	<3 (0.1)	0 (0.0)	<3 (0.1)	16 (0.4)	4 (0.1)	20 (0.5)
**G03**	*Sex hormones & modulators of genital system*	47 (1.4)	9 (0.3)	53 (1.6)	216 (5.0)	22 (0.5)	233 (5.4)
**G04**	Urologicals	<3 (0.1)	<3 (0.1)	<3 (0.1)	0 (0.0)	0 (0.0)	0 (0.0)
**H**	**Systemic hormonal preparations**	**156 (4.7)**	**9 (0.3)**	**163 (4.9)**	**186 (4.3)**	**37 (0.9)**	**220 (5.1)**
**H01**	Pituitary and hypothalamic hormones	<3 (0.1)	<3 (0.1)	<3 (0.1)	8 (0.2)	18 (0.4)	25 (0.6)
**H02**	Corticosteroids for systemic use	5 (0.1)	9 (0.3)	13 (0.4)	10 (0.2)	11 (0.3)	21 (0.5)
**H03**	*Thyroid therapy*	150 (4.5)	0 (0.0)	150 (4.5)	170 (4.0)	8 (0.2)	178 (4.2)
**J**	**Anti-infectives for systemic use**	**39 (1.2)**	**70 (2.1)**	**103 (3.1)**	**62 (1.4)**	**187 (4.4)**	**241 (5.6)**
**J01**	*Antibacterials for systemic use*	31 (0.9)	62 (1.9)	88 (2.6)	57 (1.3)	177 (4.1)	227 (5.3)
**J02**	Antimycotics for systemic use	<3 (0.1)	<3 (0.1)	<3 (0.1)	<3 (0.1)	8 (0.2)	9 (0.2)

Anatomical Therapeutic Chemical (ATC) classification index 1st and 2nd levels*		Pregnant women N = 3339			Breastfeeding women N = 4278		
		Daily use N (%)	Occasional use N (%)	Total N (%)	Daily use N (%)	Occasional use N (%)	Total N (%)
**J05**	Antivirals for systemic use	4 (0.1)	2 (0.1)	6 (0.2)	3 (0.1)	4 (0.1)	7 (0.2)
**J06**	Immune sera and immunoglobulins	0 (0.0)	<3 (0.1)	<3 (0.1)	0 (0.0)	0 (0.0)	0 (0.0)
**J07**	Vaccines**	3 (0.1)	7 (0.2)	10 (0.3)	0 (0.0)	<3 (0.1)	<3 (0.1)
**L**	**Antineoplastic and immunomodulating agents**	**11 (0.3)**	**7 (0.2)**	**18 (0.5)**	**19 (0.4)**	**3 (0.1)**	**22 (0.5)**
**L01**	Antineoplastic agents	0 (0.0)	0 (0.0)	0 (0.0)	<3 (0.1)	0 (0.0)	<3 (0.1)
**L02**	Endocrine therapy	0 (0.0)	<3 (0.1)	<3 (0.1)	<3 (0.1)	0 (0.0)	<3 (0.1)
**L03**	Immunostimulants	0 (0.0)	0 (0.0)	0 (0.0)	<3 (0.1)	0 (0.0)	<3 (0.1)
**L04**	*Immunosuppressants*	11 (0.3)	6 (0.2)	17 (0.5)	16 (0.4)	3 (0.1)	19 (0.4)
**M**	**Musculoskeletal system**	**4 (0.1)**	**24 (0.7)**	**27 (0.8)**	**56 (1.3)**	**563 (13.2)**	**613 (14.3)**
**M01**	*Antiinflammatory and antirheumatic products*	4 (0.1)	23 (0.7)	26 (0.8)	56 (1.3)	560 (13.1)	610 (14.3)
**M02**	Topical products for joint and muscular pain	0 (0.0)	<3 (0.1)	<3 (0.1)	0 (0.0)	5 (0.1)	5 (0.1)
**N**	**Nervous system**	**138 (4.1)**	**909 (27.2)**	**996 (29.8)**	**241 (5.6)**	**1384 (32.4)**	**1565 (36.6)**
**N01**	Anesthetics	<3 (0.1)	0 (0.0)	<3 (0.1)	0 (0.0)	5 (0.1)	5 (0.1)
**N02**	*Analgesics*	47 (1.4)	895 (26.8)	939 (28.1)	130 (3.0)	1371 (32.0)	1487 (34.8)
**N03**	Antiepileptics	15 (0.4)	0 (0.0)	15 (0.4)	12 (0.3)	<3 (0.1)	12 (0.3)
**N05**	Psycholeptics	15 (0.4)	20 (0.6)	35 (1.0)	14 (0.3)	26 (0.6)	40 (0.9)
**N06**	Psychoanaleptics	70 (2.1)	9 (0.3)	74 (2.2)	88 (2.1)	6 (0.1)	93 (2.2)
**N07**	Other nervous system drugs	<3 (0.1)	<3 (0.1)	<3 (0.1)	<3 (0.1)	0 (0.0)	<3 (0.1)
**P**	**Antiparasitic products, insecticides, and repellents**	**9 (0.3)**	**0 (0.0)**	**9 (0.3)**	**3 (0.1)**	**4 (0.1)**	**7 (0.2)**
**P01**	*Antiprotozoals*	9 (0.3)	0 (0.0)	9 (0.3)	3 (0.1)	<3 (0.1)	4 (0.1)
**P02**	Anthelmintics	0 (0.0)	0 (0.0)	0 (0.0)	0 (0.0)	3 (0.1)	3 (0.1)
**R**	**Respiratory system**	**417 (12.5)**	**324 (9.7)**	**671 (20.1)**	**392 (9.2)**	**324 (7.6)**	**642 (15.0)**
**R01**	Nasal preparations	82 (2.5)	66 (2.0)	144 (4.3)	99 (2.3)	69 (1.6)	161 (3.8)
**R02**	Throat preparations	0 (0.0)	8 (0.2)	8 (0.2)	0 (0.0)	7 (0.2)	7 (0.2)

Anatomical Therapeutic Chemical (ATC) classification index 1st and 2nd levels*		Pregnant women N = 3339			Breastfeeding women N = 4278		
		Daily use N (%)	Occasional use N (%)	Total N (%)	Daily use N (%)	Occasional use N (%)	Total N (%)
**R03**	Drugs for obstructive airway diseases	112 (3.4)	52 (1.6)	147 (4.4)	93 (2.2)	68 (1.6)	137 (3.2)
**R05**	Cough and cold preparations	<3 (0.1)	10 (0.3)	12 (0.4)	3 (0.1)	7 (0.2)	10 (0.2)
**R06**	*Antihistamines for systemic use*	301 (9.0)	211 (6.3)	498 (14.9)	288 (6.7)	205 (4.8)	481 (11.2)
**S**	**Sensory organs**	**26 (0.8)**	**25 (0.7)**	**50 (1.5)**	**29 (0.7)**	**22 (0.5)**	**49 (1.1)**
**S01**	*Ophthalmologicals*	23 (0.7)	22 (0.7)	44 (1.3)	28 (0.7)	15 (0.4)	42 (1.0)
**S02**	Otologicals	3 (0.1)	<3 (0.1)	4 (0.1)	<3 (0.1)	3 (0.1)	4 (0.1)
**S03**	Ophthalmological and otological preparations	0 (0.0)	<3 (0.1)	<3 (0.1)	0 (0.0)	4 (0.1)	4 (0.1)
**V**	**Various**	**4 (0.1)**	**0 (0.0)**	**4 (0.1)**	**4 (0.1)**	**0 (0.0)**	**4 (0.1)**

*The most commonly used medication groups within each ATC class (level 2) are in italics. **COVID-19 vaccines were not yet available at the time of the survey.
